# The associations of age, sex, and comorbidities with survival of hospitalized patients with coronavirus disease 2019: data from 4014 patients from a tertiary-center registry

**DOI:** 10.3325/cmj.2022.63.36

**Published:** 2022-02

**Authors:** Nevenka Piskač Živković, Marko Lucijanić, Nikolina Bušić, Ivana Jurin, Armin Atić, Ana Andrilović, Toni Penović, Iva Domić, Jelena Gnjidić, Martina Demaria, Ivan Papić, Ida Tješić-Drinković, Ivica Lukšić, Bruno Baršić

**Affiliations:** 1 Pulmonology Department, University Hospital Dubrava, Zagreb, Croatia; 2 Primary Respiratory and Intensive Care Center, University Hospital Dubrava, Zagreb, Croatia; 3 Hematology Department, University Hospital Dubrava, Zagreb, Croatia; 4 University of Zagreb, School of Medicine, Zagreb, Croatia; 5 Cardiology Department, University Hospital Dubrava, Zagreb, Croatia; 6 Department of Emergency Medicine, University Hospital Dubrava, Zagreb, Croatia; 7 Department of Physical Medicine and Rehabilitation with Rheumatology, University Hospital Dubrava, Zagreb, Croatia; 8 Pharmacy Department, University Hospital Dubrava, Zagreb, Croatia; 9 Department of Gastroenterology, Hepatology, and Clinical Nutrition, University Hospital Dubrava, Zagreb, Croatia; 10 Department of Maxillofacial Surgery, University Hospital Dubrava, Zagreb, Croatia

## Abstract

**Aim:**

To investigate how age, sex, and comorbidities affect the survival of hospitalized coronavirus disease 2019 (COVID-19) patients.

**Methods:**

We retrospectively analyzed the records of 4014 consecutive adults hospitalized for COVID-19 in a tertiary-level institution from March 2020 to March 2021.

**Results:**

The median age was 74 years. A total of 2256 (56.2%) patients were men. The median Charlson-comorbidity-index (CCI) was 4 points; 3359 (82.7%) patients had severe or critical COVID-19. A significant interaction between age, sex, and survival (*P* < 0.05) persisted after adjustment for CCI. In patients <57 years, male sex was related to a favorable (odds ration [OR] 0.50, 95% confidence interval [CI] 0.29-0.86), whereas in patients ≥57 years it was related to an unfavorable prognosis (OR 1.19, 95% CI 1.04-1.37). Comorbidities associated with inferior survival independently of age, sex, and severe/critical COVID-19 on admission were chronic heart failure, atrial fibrillation, acute myocardial infarction, acute cerebrovascular insult, history of venous thromboembolism, chronic kidney disease, major bleeding, liver cirrhosis, mental retardation, dementia, active malignant disease, metastatic malignant disease, autoimmune/rheumatic disease, bilateral pneumonia, and other infections on admission.

**Conclusion:**

Among younger patients, female sex might lead to an adverse prognosis due to undisclosed reasons (differences in fat tissue distribution, hormonal status, and other mechanisms). Patient subgroups with specific comorbidities require additional considerations during hospital stay for COVID-19. Future studies focusing on sex differences and potential interactions are warranted.

Coronavirus disease 2019 (COVID-19) caused by severe acute respiratory syndrome coronavirus 2 (SARS-CoV-2) is a systemic disease presenting with predominantly respiratory symptoms. Up to 15%-20% of affected individuals develop high inflammatory state and severe intensity of symptoms requiring hospital admission in (1). Age and comorbidities were among the earliest recognized clinical risk factors for adverse disease course, and have consistently been shown to affect the severity of presentation and survival of COVID-19 patients. Unfavorable disease course has been especially associated with chronic metabolic comorbidities, such as arterial hypertension, diabetes mellitus, hyperlipoproteinemia, and obesity (2-7). Charlson comorbidity index (CCI), a summary measure of comorbidities validated as a prognostic tool in a number of chronic and malignant diseases (8,9), has also been associated with an adverse COVID-19 clinical course (10-15).

Since elderly patients who are more frail and more prone to more severe COVID-19 are also more burdened with comorbidities, in some patients it is almost impossible to distinguish whether clinical deterioration and adverse clinical outcomes are attributable to COVID-19 or to prior comorbidities. Higher inflammatory state associated with COVID-19 might lead to clinical decompensation of chronic comorbidities, and *vice versa,* prior comorbidities and elevated baseline inflammatory state might predispose to more severe COVID-19. Due to this complex relationship and the need for better understanding how and to what extent particular comorbidities affect the survival of COVID-19 patients, we aimed to investigate the associations of age, sex, and comorbidities with survival in a large cohort of hospitalized COVID-19 patients treated in our institution. We hypothesized that older age, male sex, and higher comorbidity burden were associated with higher death rates.

## Patients and methods

This retrospective, registry-based study used data from the hospital Registry obtained through the analysis of written and electronic medical records of all COVID-19 patients admitted to University Hospital Dubrava. Our institution was repurposed to become a regional tertiary COVID-19 center. Among 4102 hospital admissions, we retrospectively analyzed the records of 4014 consecutive adults who had an index hospital admission because of COVID-19. Patients were treated from March 2020 to March 2021 and they all completed their index hospital admission. All patients had a positive polymerase chain reaction or antigen COVID-19 test before hospital admission. Patients were treated according to the contemporary guidelines. The study was approved by the University Hospital Dubrava Review Board (2021/2503-04).

COVID-19 disease severity on admission was graded as mild, moderate, severe, or critical based on the World Health Organization (WHO) and national guidelines (16,17). Obesity was defined as body mass index above 30. Comorbidities were assessed as individual entities and summarized by using CCI (18). In-hospital mortality and survival at 30 days from hospital admission were assessed as clinical outcomes.

### Statistical analysis

The normality of distribution of numerical variables was tested by using the Kolmogorov-Smirnov test. Numerical variables are presented as median and interquartile range (IQR), and categorical variables as frequencies and percentages. Differences in numerical variables between the subgroups were assessed by using the Mann-Whitney U test and Kruskal-Wallis ANOVA test with *post-hoc* analysis by the Conover test. Trends of increase in age, CCI, and male sex across COVID-19 severity categories were assessed by using the Jonckheere-Terpstra test for trend and Χ^2^ test for trend. The associations of investigated parameters with in-hospital survival were assessed by using the logistic regression analysis. For the assessment of independent associations of specific comorbidities with COVID-19 severity and survival, a model was built by using the backward approach with *P* < 0.05 and *P* > 0.1 criteria for variable inclusion and removal, respectively. Receiver-operating characteristic curve analysis was used to establish the optimal cut-off level for in-hospital mortality discrimination for age. Thirty-day survival estimates were based on the Kaplan-Meier method and univariate survival analyses, and were performed by using the log-rank test. *P* values <0.05 were considered statistically significant. All analyses were performed with MedCalc statistical software, version 20.006 (MedCalc Software Ltd, Ostend, Belgium).

## Results

### An overview of patient population

We enrolled 4014 patients (2256 or 56.2% men) with the index hospitalization for COVID-19 infection. The median age was 74 years (IQR 64-82). The median disease duration at hospital admission was 5 days (IQR 1-9). COVID-19 severity at hospital admission was mild in 449 (11.2%), moderate in 206 (5.1%), severe 2761 (68.8%), and critical in 598 (14.9%) patients. Severe and critical COVID-19 symptoms were present in 3359 (82.7%) of patients. A total of 3531 (88%) patients had pneumonia and 3265 (81.3%) required oxygen supplementation. The median CCI was 4 points (IQR 3-6). The frequencies of specific comorbidities are presented in Table 1. Overall, 1428 (35.6%) patients died during the index hospital stay, with a 30-day survival rate of 65.4%.

**Table 1:** Frequency and prognostic associations of specific comorbidities (crude and age- and sex-adjusted OR estimates for in-hospital mortality with 95% CIs)

		OR with 95% CI for in-hospital mortality	
Comorbidity	Frequency (%) (N = 4014)	crude	age- and sex-adjusted
Arterial hypertension	2771 (69)	1.5 (1.3-1-74); *P* < 0.001	0.95 (0.81-1.1); *P* = 0.532
Diabetes mellitus	1201 (29.9)	1.27 (1.11-1.46); *P* < 0.001	1.16 (1.0-1.34); *P* = 0.049
Hyperlipoproteinemia	954 (23.8)	1.19 (1.03-1.38); *P* = 0.022	1.09 (0.94-1.28); *P* = 0.259
Obesity	1069 (26.6)	0.91 (0.79-1.06); *P* = 0.233	1.17 (1.0-1.37); *P* = 0.045
Metabolic syndrome	799 (19.9)	1.17 (1.0-1.38); *P* = 0.050	1.15 (0.98-1.36); *P* = 0.094
Chronic heart failure	649 (16.2)	2.15 (1.82-2.55); *P* < 0.001	1.56 (1.31-1.87); *P* < 0.001
Atrial fibrillation	721 (18)	2.14 (1.81-2.51); *P* < 0.001	1.35 (1.14-1.61); *P* < 0.001
Coronary artery disease	613 (15.3)	1.65 (1.39-1.96); *P* < 0.001	1.34 (1.11-1.61); *P* = 0.002
Peripheral artery disease	281 (7)	1.74 (1.37-1.23); *P* < 0.001	1.40 (1.08-1.8); *P* = 0.010
History of myocardial infarction	366 (9.1)	11.59 (1.28-1.97); *P* < 0.001	1.31 (1.05-1.65); *P* = 0.018
History of cerebrovascular insult	469 (11.7)	1.64 (1.35-1.99); *P* < 0.001	1.25 (1.02-1.54); *P* = 0.028
Acute myocardial infarction	68 (1.7)	2.63 (1.62-4.29); *P* < 0.001	2.35 (1.4-3.93); *P* = 0.001
Acute cerebrovascular insult	111 (2.8)	1.74 (1.19-2.54); *P* = 0.004	1.53 (1.04-2.27); *P* = 0.033
History of venous thromboembolism	193 (4.8)	1.77 (1.32-2.37); *P* < 0.001	1.79 (1.33-2.44); *P* < 0.001
VTE on admission	68 (1.7)	0.95 (0.59-1.63); *P* = 0.954	1.12 (0.66-1.9); *P* = 0.671
Chronic kidney disease	498 (12.4)	1.86 (1.54-2.24); *P* < 0.001	1.54 (1.26-1.87); *P* < 0.001
Chronic hemodialysis	76 (1.9)	0.99 (0.62-1.6); *P* = 0.993	1.15 (0.7-1.9); *P* = 0.573
Major bleeding	129 (3.2)	2.77 (1.94-3.97); *P* < 0.001	2.82 (1.93-4.14); *P* < 0.001
GERD/Ulcer disease	566 (14.1)	1.24 (1.04-1.49); *P* = 0.019	1.15 (0.95-1.39); *P* = 0.149
Inflammatory bowel disease	46 (1.1)	0.87 (0.47-1.63); *P* = 0.672	1.29 (0.66-2.51); *P* = 0.459
Chronic liver disease	110 (2.7)	1.42 (0.97-2.08); *P* = 0.075	2.11 (1.41-3.18); *P* < 0.001
Liver cirrhosis	49 (1.2)	2.66 (1.49-4.71); *P* < 0.001	3.81 (2.08-6.96); *P* < 0.001
Epilepsy	112 (2.8)	1.09 (0.74-1.61); *P* = 0.666	1.49 (0.98-2.27); *P* = 0.064
Mental retardation	45 (1.1)	0.82 (0.43-1.54); *P* = 0.530	2.8 (1.39-5.66); *P* = 0.004
Schizophrenia	60 (1.5)	0.71 (0.4-1.25); *P* = 0.239	0.87 (0.48-1.58); *P* = 0.645
Dementia	829 (20.7)	3.08 (2.63-3.6); *P* < 0.001	1.89 (1.59-2.24); *P* < 0.001
Active malignant disease	429 (10.7)	1.61 (1.31-1.97); *P* < 0.001	1.88 (1.51-2.33); *P* < 0.001
Metastatic malignant disease	280 (7)	1.87 (1.47-2.39); *P* < 0.001	2.37 (1.82-3.07); *P* < 0.001
History of malignant disease	718 (17.9)	1.44 (1.22-1.69); *P* < 0.001	1.44 (1.22-1.72); *P* < 0.001
Thyroid disease	371 (9.2)	0.97 (0.78-1.22); *P* = 0.821	0.98 (0.77-1.25); *P* = 0.877
Autoimmune/rheumatic disease	174 (4.3)	1.08 (0.79/1.48); *P* = 0.616	1.49 (1.07-2.09); *P* = 0.017
Asthma	119 (3)	0.81 (0.55-1.2); *P* = 0.301	1.13 (0.74-1.72); *P* = 0.576
COPD	286 (7.1)	1.38 (1.08-1.76); *P* = 0.009	1.12 (0.87-1.44); *P* = 0.381
Transplanted organ	43 (1.1)	0.97 (0.52-1.82); *P* = 0.924	1.84 (0.94-3.6); *P* = 0.076
Pneumonia	3531 (88)	14.57 (9.36-22.68); *P* < 0.001	12.01 (7.67-18.82); *P* < 0.001
Bilateral pneumonia	2600 (63.8)	2.49 (2.16-2.89); *P* < 0.001	2.56 (2.19-2.99); *P* < 0.001
Other infection on admission	587 (14.6)	1.58 (1.32-1.88); *P* < 0.001	1.36 (1.13-1.64); *P* = 0.002

*Abbreviations: OR – odds ratio; CI – confidence interval; VTE – venous thromboembolism; GERD – gastroesophageal reflux disease; COPD – chronic obstructive pulmonary disease.

### Associations of age, sex, and Charlson comorbidity index with COVID-19 severity on admission

Patients presenting with more severe COVID-19 on admission were significantly more likely to be of older age and male sex, and to have higher CCI (*P* < 0.05 for difference and for increasing trend across higher COVID-19 severity categories). Similarly, patients with severe or critical COVID-19 on admission were more likely to be older (median 74 vs 69 years; *P* < 0.05) and of male sex (57.2% vs 51.3%; *P* < 0.05), and to have higher CCI (median 5 vs 4; *P* < 0.05) than patients with non-severe COVID-19.

### Associations of age, sex and Charlson comorbidity index with survival

**Figure 1:**
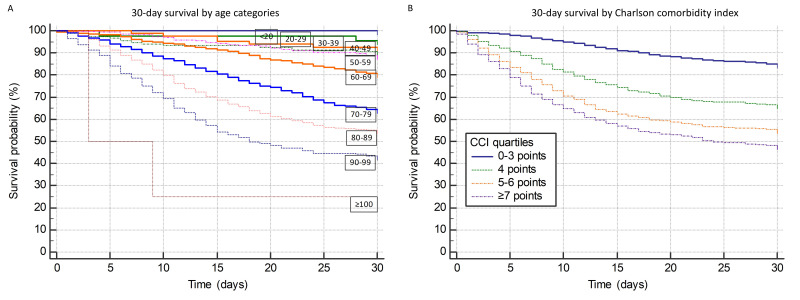
Survival at thirty days from admission stratified by (**A**) 10-years age groups and (**B**) Charlson comorbidity index (CCI) quartiles.

Higher age was significantly associated with higher in-hospital mortality when used as a continuous variable (odds ratio [OR] 1.06, 95% confidence interval [CI] 1.05-1.07, *P* < 0.05) and when stratified to 10-year categories (OR 1.7, 95% CI 1.61-1.8, *P* < 0.05). In-hospital mortality rates associated with specific age categories were 0/6 (0%) for <20 years, 3/43 (7%) for 20-29 years, 6/80 (7.5%) for 30-39 years, 17/181 (9.4%) for 40-49 years, 63/409 (15.4%) for 50-59 years, 193/810 (23.8%) for 60-69 years, 449/1117 (40.2%) for 70-79 years, 554/1129 (49.1%) for 80-89 years, 140/235 (59.6%) for 90-99 years and 3/4 (75%) for 100 years and older. Thirty-day survival curves stratified by age categories are shown in Figure 1A. The largest difference in mortality was detected at the age cut-off of >70 years (47.1% vs 19%: OR 3.8, 95% CI 3.28-4.41, *P* < 0.05).

**Figure 2:**
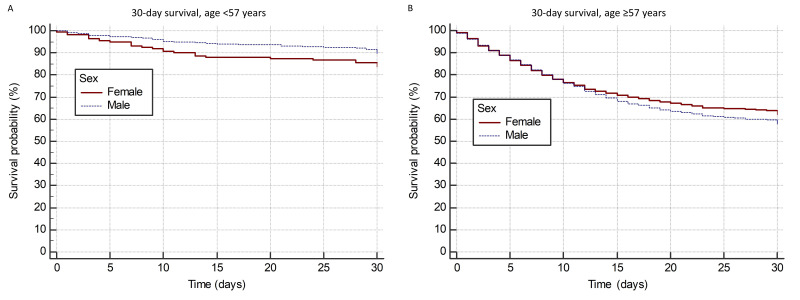
Survival at thirty days from admission stratified by sex for patients (**A**)<57 years and (**B**)≥57 years.

**Figure 3:**
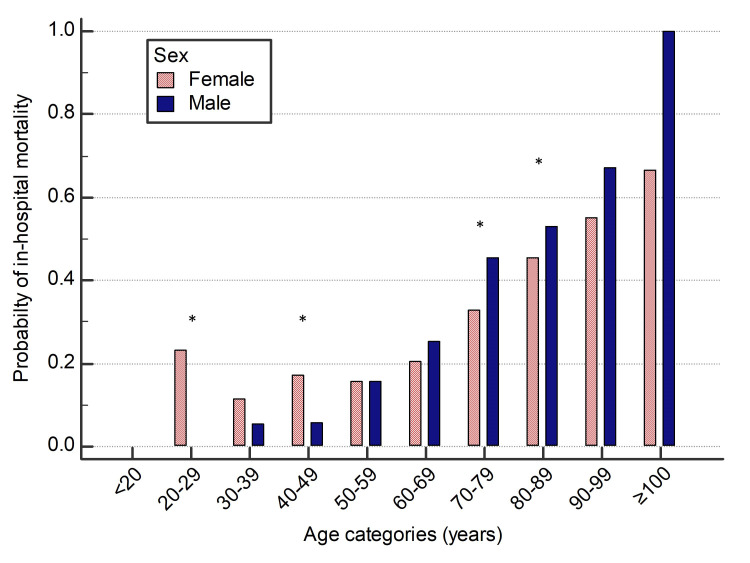
Age-stratified and sex-stratified in-hospital mortality rates. Asterisk indicates significant sex differences in mortality in age groups.

In the overall sample and unadjusted analyses, sex was not significantly associated with survival (0.499). However, since female patients were significantly older than male (median 78 vs 71 years; *P* < 0.05), after adjusting for age, male patients had significantly worse survival (OR 1.41, 95% CI 1.23-1.63, *P* < 0.05) independently of age (OR 1.06, 95% CI 1.06-1.07, *P* < 0.05), which showed that the association of sex with survival in the overall cohort was masked due to age differences. Moreover, age, sex, and survival significantly interacted (*P* < 0.05). The effect of sex on survival changes depended on the age of patients. This phenomenon persisted after adjusting for CCI and was present independently of CCI (interaction term age*sex OR 1.0, 95% CI 1.0-1.01, *P* < 0.05; CCI OR 1.31, 95% CI 1.28-1.35, *P* < 0.05). In patients younger than 57 years, female sex was associated with worse prognosis (OR for male sex 0.50, 95% CI 0.29-0.86, *P* < 0.05; Figure 2A), whereas in patients ≥57 years male sex was associated with worse prognosis (OR 1.19, 95% CI 1.04-1.37, *P* < 0.05; Figure 2B) (Figure 3).

CCI was significantly associated with worse survival when used as a continuous variable (OR 1.31, 95% CI 1.28-1.35, *P* < 0.05), with the risk of death increasing 30% for every additional point, as well as when stratified to quartiles (Figure 1B). The relationship of CCI with a worse outcome persisted after adjusting analyses for age, sex, and disease severity at presentation (CCI OR 1.24, 95% CI 1.19-1.28, *P* < 0.05; age OR 1.04, 95% CI 1.03-1.05, *P* < 0.05; male sex OR 1.2, 95% CI 1.03-1.4, *P* < 0.05, COVID severity grade OR 4.42, 95% CI 3.78-5.17, *P* < 0.05).

### Associations of specific comorbidities with survival

Since specific comorbidities were more prevalent in different age groups and sex groups compared with general cohort, in Table 1 we present crude and age-adjusted and sex-adjusted odds ratios for in-hospital mortality associated with specific comorbidities. All comorbidities with either crude or age-adjusted and sex-adjusted univariately significant associations with survival, together with age, sex, and COVID-19 severity on admission, were included in the logistic regression model building process using the backward approach (Table 2). The comorbidities that remained mutually independently associated with inferior survival were older age (OR 1.06), male sex (OR 1.3), chronic heart failure (OR 1.34), atrial fibrillation (OR 1.27), acute myocardial infarction (OR 2.37), acute cerebrovascular insult (OR 1.95), history of venous thromboembolism (OR 1.48), chronic kidney disease (OR 1.39), major bleeding (OR 3.43), liver cirrhosis (OR 4.89), mental retardation (OR 2.97), dementia (OR 1.86), active malignant disease (OR 1.66), metastatic malignant disease (OR 2.22), autoimmune/rheumatic disease (OR 1.58), bilateral pneumonia (OR 2.08), other infections on admission (OR 1.31), and severe or critical COVID-19 on admission (OR 13.81).

**Table 2:** Logistic regression model investigating mutually independent associations of age, sex, coronavirus disease 2019 (COVID-19) severity, and specific comorbidities with in-hospital mortality

Variable	P	Adjusted odds ratio with 95% confidence interval
Age (years)	0.001	1.06 (1.05-1.06)
Male sex	0.002	1.3 (1.1-1.52)
Arterial hypertension	0.050	0.83 (0.7-1.0)
Chronic heart failure	0.007	1.34 (1.08-1.65)
Atrial fibrillation	0.019	1.27 (1.04-1.54)
Coronary artery disease	0.083	1.21 (0.98-1.51)
Peripheral artery disease	0.054	1.34 (0.99-1.8)
Acute myocardial infarction	0.005	2.37 (1.3-4.32)
Acute cerebrovascular insult	0.004	1.95 (1.24-3.07)
History of venous thromboembolism	0.025	1.48 (1.05-2.07)
Chronic kidney disease	0.004	1.39 (1.11-1.75)
Major bleeding	0.001	3.43 (2.24-5.25)
Liver cirrhosis	0.001	4.89 (2.44-9.8)
Mental retardation	0.003	2.97 (1.45-6.09)
Dementia	0.001	1.86 (1.54-2.25)
Active malignant disease	0.008	1.66 (1.14-2.41)
Metastatic malignant disease	0.001	2.22 (1.41-3.5)
Autoimmune/rheumatic disease	0.013	1.58 (1.1-2.26)
Bilateral pneumonia	0.001	2.08 (1.73-2.48)
Other infection on admission	0.013	1.31 (1.06-1.62)
Severe or critical COVID-19 on admission	0.001	13.81 (8.96-21.28)

## Discussion

Our real-life data from a large tertiary center registry confirmed that age, sex, and comorbidity burden were significantly associated with more severe COVID-19 on admission and worse survival of hospitalized COVID-19 patients independently of one other. The important novel finding was that age and sex might moderate each other’s effect on the survival of hospitalized COVID-19 patients. This phenomenon was independent of CCI and thus might not be directly attributable to differences in comorbidity burden. Older age affected the survival of male COVID-19 patients more profoundly, with the difference in survival becoming skewed in favor of women after the fifth decade of life. It is unknown why women in younger age groups have worse prognosis. We speculate the reasons could be different fat tissue body composition, fat tissue distribution, and hormonal differences between sexes, which diminish in older age groups.

Since specific comorbidities are typically present in age and sex subgroups that differ from the overall cohort average, their associations with survival were either masked or more pronounced in unadjusted analyses. Arterial hypertension, which was the most common comorbidity, lost its association with survival after adjustment for age and sex. Patients with arterial hypertension and hyperlipoproteinemia were older than the rest of the cohort and the detrimental association of these conditions with survival was at least in part caused by older age. The opposite was true in patients with obesity, which was recognized as a negative prognostic parameter after age and sex adjustments. Age, sex, and comorbidity burden substantially affected the outcomes of hospitalized COVID-19 patients. Hence, differences in these parameters in different published cohorts make straightforward indirect comparisons difficult.

Our data confirm previously reported negative prognostic value of high comorbidity burden as assessed with CCI in patients with COVID-19 (10-13). CCI, a composite measure of comorbidity burden, is as practical tool to assess the contribution of comorbidities to COVID-19 prognosis (19-21). It was included in some of the COVID-19 prognostic scores, such as Veterans Health Administration COVID-19 Index for COVID-19 Mortality (22).

It is of particular interest that specific acute and chronic comorbidities contribute to higher in-hospital mortality independently of age, sex, and COVID-19 severity. Severe or critical COVID-19 on admission most strongly affects survival, and bilateral pneumonia independently increases the risk of death for about two times. These are followed by the presence of liver cirrhosis and major bleeding. Survival was also substantially affected by cognitive impairment (due to patients' inability to fully cooperate with medical procedures), acute cardiovascular complications, and chronic cardiovascular diseases. A history of venous thromboembolism, which might imply intrinsic pro-thrombotic condition, was also recognized as an important independent predictor of worse survival. Active and metastatic malignancy, autoimmune/rheumatic disease, and other infections on admission were also significantly independently associated with worse survival.

Limitations of this study are retrospective design and single-center experience. Our results are representative of tertiary-level hospital experience and might not be generalizable to other contexts of medical care for COVID-19 patients. However, this is one of the largest published cohorts of hospitalized COVID-19 patients so far, providing the opportunity to thoroughly investigate associations and relationships of age, sex, comorbidities, and survival. Our study highlights the fact that age and sex differences might mask the relationship of specific parameters with survival, as well as that age and sex might mutually moderate the associations with survival among hospitalized COVID-19 patients. Future studies focusing on sex differences and potential interactions are warranted.

In conclusion, patient subgroups with specific comorbidities require additional considerations during hospital admission for COVID-19. High-risk comorbidities that are independently associated with worse survival should be recognized early and appropriately treated.
